# Berberine-targeted miR-21 chemosensitizes oral carcinomas stem cells

**DOI:** 10.18632/oncotarget.20723

**Published:** 2017-09-08

**Authors:** Che-Yi Lin, Pei-Ling Hsieh, Yi-Wen Liao, Chih-Yu Peng, Ming-Yi Lu, Ching-Hsuan Yang, Cheng-Chia Yu, Chia-Ming Liu

**Affiliations:** ^1^ Department of Oral and Maxillofacial Surgery, Chi Mei Hospital, Tainan, Taiwan; ^2^ Institute of Oral Sciences, Chung Shan Medical University, Taichung, Taiwan; ^3^ School of Dentistry, Chung Shan Medical University, Taichung, Taiwan; ^4^ Department of Dentistry, Chung Shan Medical University, Taichung, Taiwan

**Keywords:** oral squamous cell carcinomas, miR-21, berberine

## Abstract

Cancer recurrence and chemoresistance are two major obstacles to the treatment of oral squamous cell carcinomas (OSCC). And cancer stem cells (CSCs) have been found to possess tumor initiating, self-renewal and metastasis abilities, resulting in the relapse and chemoresistance of OSCC. In the present study, we investigated the anti-CSCs effect of berberine, a phenanthrene alkaloid isolated from the Berberis genus. Our results demonstrated that berberine dose dependently downregulated the oncogenicity *in vitro*, including ALDH1 activity, self-renewal property, and colony formation and invasion abilities as well as potentiated chemosensitivity of OSCC-CSCs. In addition, tumor growth in mice was attenuated after oral gavage treatment of berberine. We showed that the expression of miR-21 was suppressed following administration of berberine in OSCC-CSCs. And inhibition of endogenous miR-21 reduced the characteristics of CSCs, including self-renewal, migration, invasion capabilities and ALDH1 activity. Taken together, we demonstrated the anti-CSC effect of berberine in oral cancer and its potential to serve as adjuvant to traditional chemotherapy to improve treatment effect.

## INTRODUCTION

Oral squamous cell carcinoma (OSCC) represents one of the most common cancers with increased annual incidence [1] and has a high propensity for treatment failure. Resistance to chemo/radiotherapy and recurrence are major causes of mortality in patients with OSCC [2], and there is still a significant number of patients suffer from recurrent cancer after chemotherapy [3]. A small subset of cells, often termed cancer stem cells (CSCs), have been considered as key contributors to drug resistance, tumor relapse and metastasis in OSCC [4]. Hence, agents that eliminate and sensitize the chemoresistant CSCs are extremely crucial to enhance the current treatment effectively.

Berberine is a phenanthrene alkaloid isolated from the roots and bark of plants from the Berberis genus (Berberidaceae family). It has traditionally been used in Oriental medicine and has various biological activities, including anti-microbial [5, 6], anti-inflammatory [7] and antioxidant [8, 9] properties. Recent studies have focused on its anti-tumor effects, including anti-metastasis, apoptosis and autophagy induction and epithelial-to-mesenchymal transition (EMT) suppression in a wide range of tumor cell types [10–18]. And a number of reports have demonstrated that it induces apoptosis in oral cancer cells [19, 20]. Moreover, it has been shown to diminish CSCs and down-regulate stem cell-associated genes in the pancreatic cancer cell lines [21]. In addition, it was reported to sensitize ovarian cancer cell to cisplatin [22]. Hence, its anti-tumor effect on OSCC-CSCs is worth investigating.

MicroRNAs (miRNAs) are small noncoding RNAs regulating the gene expression at the post-transcriptional level and have been found to be involved in various biological processes [23–25]. In particular, miR-21 has been indicated as one of the most upregulated miRNAs in OSCC [26] and shown to confer resistance to chemotherapy, such as 5-fluorouracil (5-FU) and cisplatin, in hepatocellular carcinoma [27], neuroblastoma [28], colorectal [29], and gastric cancers [30]. Furthermore, it has been shown that berberine suppresses multiple myeloma cell growth by down-regulating miR-21 expression [31]. Accordingly, it is also imperative to elucidate whether berberine and miR-21 are associated with sensitizing the chemoresistant CSCs and eliciting anti-OSCC activity.

In this study, we evaluated the effect of berberine on cell viability, ALDH1 activity, self-renewal, colony formation, invasion abilities *in vitro* and tumorigenecity *in vivo*. Additionally, we found that administration of berberine enhanced the tumor sensitization to chemotherapy. Besides, our results demonstrated that administration of berberine decreased the expression of miR-21 in a dose-dependent manner. And inhibition of miR-21 suppressed the features of CSCs, including self-renewal, migration, invasion capacities and the expression of CSC marker. These data indicate that miR-21 may play a pivotal role in the anti-CSCs property of berberine. In conclusion, we demonstrated the tumor suppressive and chemosensitizing effect of berberine in OSCC-CSCs via regulation of miR-21.

## RESULTS

### The cytotoxic effect of berberine on OSCC cancer stem cells (OSCC-CSCs)

**Figure 1:**
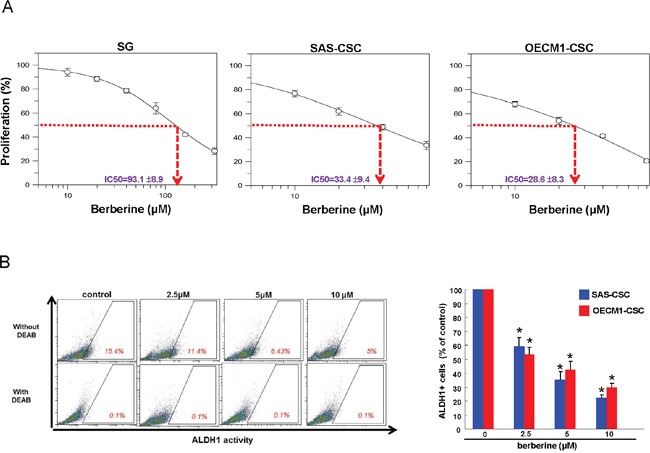
Cell viability and ALDH1 activity of OSCC-CSCs after berberine treatment **(A)** Cytotoxic effect was determined by MTT assay. Berberine markedly inhibited the cell survival of OSCC-CSCs in a dose-dependent manner without causing damage to normal cells; **(B)** ALDH1 enzymatic activity was gradually eliminated in OSCC-CSCs as the concentration of berberine increased. Values were expressed as mean ±SD. * *p* <.05 compared to control.

MTT assay was used to determine the cell viability of two OSCC-CSCs (SAS and OECM-1) and normal human gingival epithelioid cell line (SG) after treatment with increasing concentrations of berberine. As shown in Figure 1A, berberine markedly suppressed the cell survival of two OSCC-CSCs without causing toxic damage to normal SG cells. The cytotoxicity data indicated a dose response relationship with respect to berberine and OSCC-CSCs viability.

### ALDH1 enzymatic activity in OSCC-CSCs was repressed by berberine

Aldehyde dehydrogenase (ALDH1) enzymatic activity has been shown to be a highly selective marker for CSCs in HNSCC [32]. And it has been suggested that elevated ALDH1 level correlated with local recurrence of OSCC and there were more ALDH1 expressing cells existed in cisplatin-surviving cells [33]. In this study, flow cytometry analysis showed that ALDH1 activity was gradually attenuated in both OSCC-CSCs along with the increase in berberine concentration (Figure 1B), indicating that berberine may be able to suppress the characteristics of OSCC-CSCs.

### Reduced self-renewal and oncogenicity abilities in OSCC-CSCs by berberine

**Figure 2:**
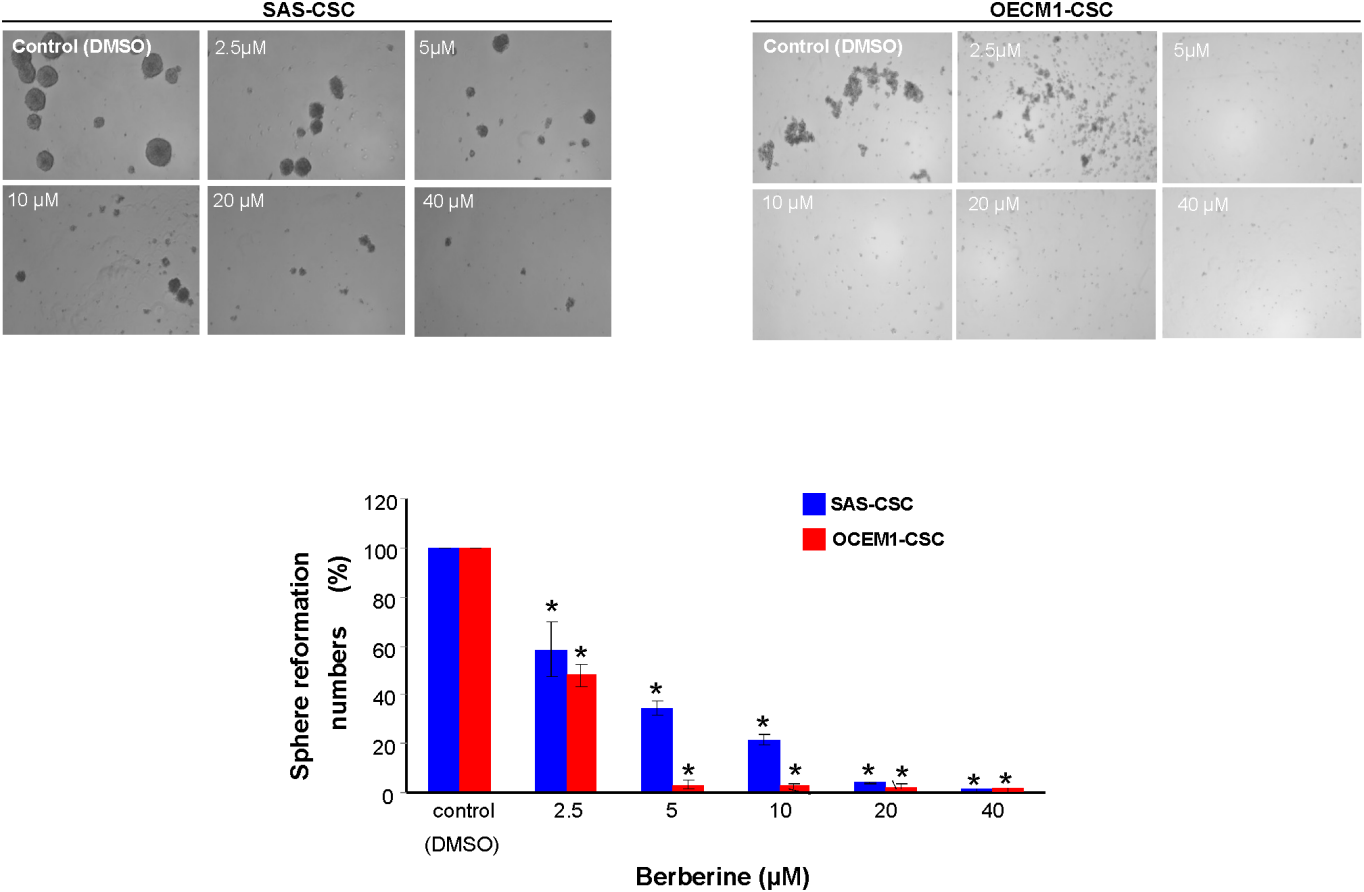
Inhibition of self-renewal property in OCSC after berberine administration Secondary sphere formation ability of berberine-treated cells was examined. The percentage of the sphere formation was suppressed by berberine in both OSCC-CSCs. Values were expressed as mean ±SD. * *p* <.05 compared to DMSO control.

**Figure 3:**
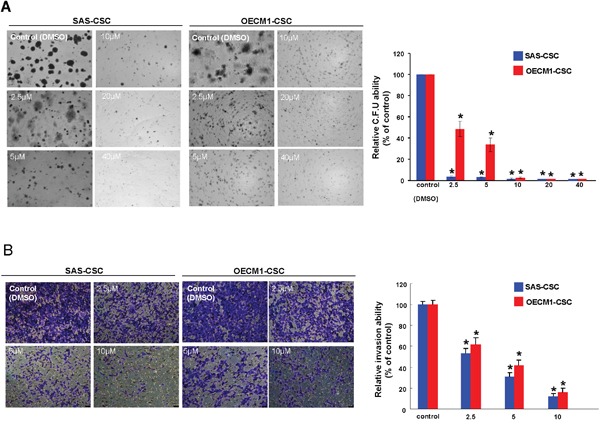
Oncogenicity abilities were hindered by berberine **(A)** Colony formation capacity in OSCC-CSCs was reduced by administration of berberine; **(B)** The invasion potential of OSCC-CSCs was interfered by berberine in a dose-dependent fashion. Values were expressed as mean ±SD. * *p* <.05 compared to DMSO control.

One of the CSCs hallmarks is self-renewal capacity and sphere reformation over serial passages is gold standard method to evaluate this property [34]. Our data showed that self-renewal ability in both OSCC-CSCs was inhibited dose-dependently following administration of berberine (Figure 2). Also, CSCs are known to be equipped with the tumor initiating property and metastatic behavior, both of which are essential for orchestrating field cancerization and tumor recurrence [35]. The result of colony formation assay showed that anchorage-independent growth capability was hindered by the increase in berberine (Figure 3A). To assess the metastatic potential, we treated cells with various concentration of berberine followed by examination of invasion ability using transwell. We observed a dose-dependent effect of berberine on the invasion capacities of OSCC-CSCs (Figure 3B). Altogether, these results clearly demonstrated that berberine exerted a pronounced anti-CSC effect.

### Enhanced chemosensitivity in OSCC-CSCs by berberine

**Figure 4:**
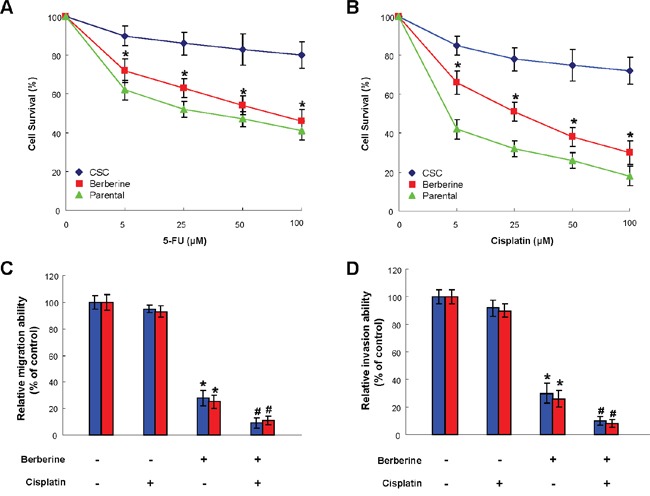
Use of berberine to potentiate chemotherapy Cell viability data suggested that chemoresistance to 5-FU **(A)** cisplatin **(B)** or were more obvious in OSCC-CSCs compared to parental OSCC cells, while chemosensitivity in OSCC-CSCs was enhanced by berberine treatment. The relative migration **(C)** and invasion **(D)** capacities of OSCC-CSCs were improved after berberine alone treatment or in conjunction with cisplatin. Values were expressed as mean ±SD. * *p* <.05 compared to no treatment control.

The chemoresistant CSCs has been implicated in the cancer recurrence following conventional therapy [36], hence it is crucial to evaluate whether berberine possess the potential to enhance the efficacy of chemotherapy. As expected, drug-resistance was more prominent in OSCC-CSCs in comparison with parental OSCC cells (Figure 4). Nonetheless, the sensitivity of OSCC-CSCs to both 5-FU and cisplatin was dramatically improved in conjunction with berberine (Figure 4). Collectively, these findings showed that berberine may serve as an adjunct to traditional chemotherapeutic agents.

### Administration of berberine exerts a suppressive effect on tumor growth *in vivo*

**Figure 5:**
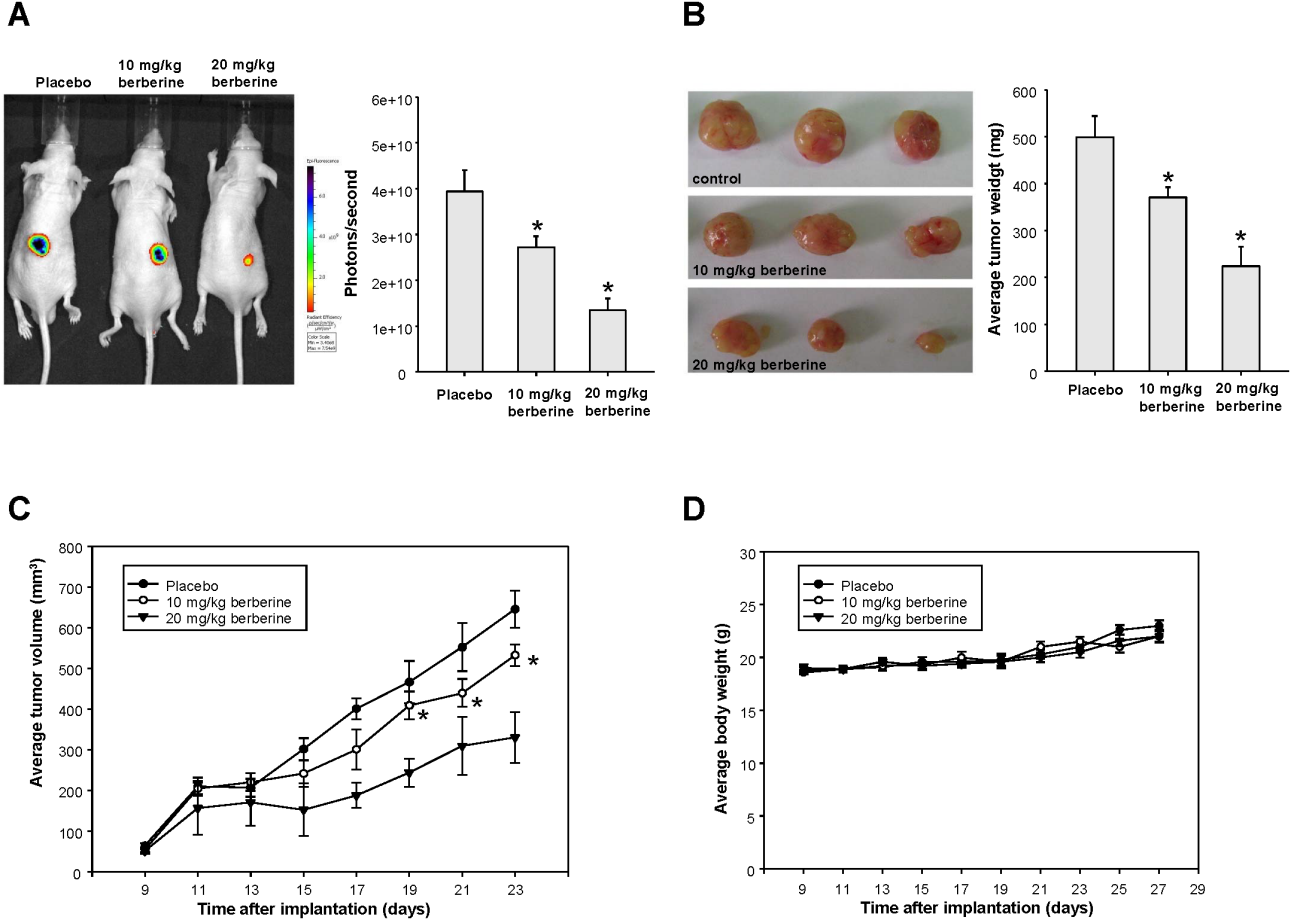
The anti-tumor effect of berberine *in vivo* The bioluminescent signal **(A)** and the excised tumor **(B)** both showed that tumor size was reduced by berberine treatment; **(C)** Data from IVIS Spectrum imager indicated that tumor growth was significantly delayed after delivery of berberine in mice bearing OSCC-CSCs xenografts; **(D)** Body weight of the mice was not affected by administration of berberine. Values were expressed as mean ±SD. * *p* <.05 compared to placebo group.

After examining the anti-CSCs effect of berberine *in vitro*, we investigated its influence on tumorigenicity in xenotransplantation model. Immunocompromised mice bearing OSCC-CSCs xenografts were assigned to receive oral administration of berberine or vehicle followed by tumor volume assessment. The bioluminescent signal in photons per second from the IVIS Spectrum imager were in accord with the volume of the excised tumors (Figure 5A and 5B), and both of them displayed a dose dependent reduction of tumor. We demonstrated that the tumor growth was significantly delayed after treatment of berberine (Figure 5C) without causing body weight loss (Figure 5D).

### The anti-CSC effect of berberine is via down-regulation of miR-21

**Figure 6:**
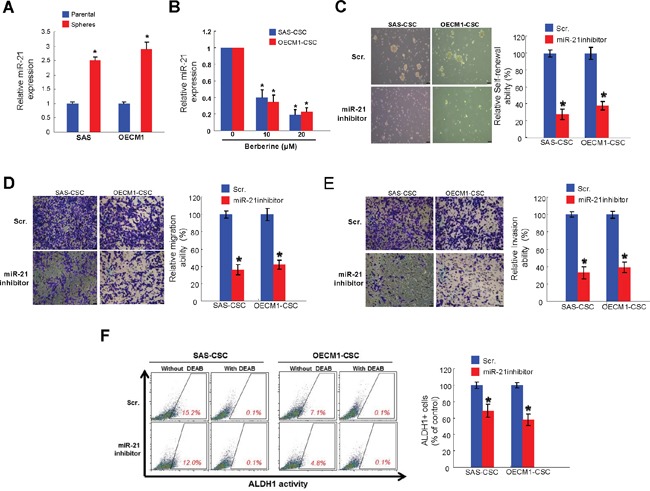
Berberine-mediated anti-tumor response is via down-regulation of miR-21 **(A)** Relative of miR-21 expression in OSCC-CSCs and OSCC cell lines was examined by miRNAs real-time RT-PCR analysis. **(B)** The expression of miR-21 was reduced after berberine in both OSCC-CSCs; Inhibition of miR-21 using sponges abrogated the self-renewal **(C)**, migration **(D)** and invasion **(E)** abilities of OSCC-CSCs; **(F)** The ALDH1 enzymatic activity was reduced by miR-21 inhibitor. Values were expressed as mean ±SD. * *p* <.05 compared to control group.

miRNAs have recently been linked to regulate cancer stemness in OSCC. A significant increase in oncogenic miR-21 expression was in OSCC-CSCs relative to parental OSCC cells (Figure 6A). The expression of miR-21 in two OSCC-CSCs was reduced after berberine in a dose-dependent fashion (Figure 6B). We showed that inhibition of endogenous miR-21 using sponges to create loss-of-function phenotype led to reduced self-renewal (Figure 6C), migration (Figure 6D) and invasion (Figure 6E) capabilities. Additionally, the ALDH1 activity was suppressed in both OSCC-CSCs after knockdown of miR-21 (Figure 6F). And these data indicated that the repressed hallmarks of CSCs may be associated with the reduced miR-21 following berberine treatment.

## DISCUSSION

Drug resistance and cancer recurrence remain as major causes of death in patients with OSCC [2]. Among various factors regulating cancer aggressiveness, miR-21 has been recently indicated as an important contributing factor. It has not only been identified as one of the most upregulated miRNAs in OSCC [26] but also shown to confer resistance to chemotherapy in various cancers [27–30]. Several studies have suggested that overexpression of miR-21 dramatically reduced the therapeutic efficacy of chemotherapy via downregulating the expression of human mutS homolog 2 [29, 37] or PTEN/ Akt pathway [28, 30], whereas inhibition of miR-21 reversed this effect and therefore reduced oncogenicity [30, 37]. In accordance with these findings, we demonstrated that downregulation of miR-21 by berberine reduced the expression of CSC marker and inhibited the self-renewal, migration and invasion capabilities of OSCC-CSCs (Figure 6).

As a matter of fact, berberine has been proven to inhibit the growth of various cancers through suppression of EMT [14, 15], induction of apoptosis [11, 18–20, 38–43] and autophagy [16, 17], and cell cycle arrest [11, 41, 42, 44] as well as to reduce the metastatic potential via inhibition of COX-2/ PGE_2_ [12] or AMPK/ ERK pathways [13]. Berberine was also reported to diminish the CSCs [21], which play a crucial role in relapse of cancer and metastasis. In the current study, we further showed the direct inhibitory effect of berberine on OSCC-CSCs via targeting miR-21, resulting in attenuated tumor growth *in vivo*.

Most importantly, we demonstrated that berberine potentiated chemotherapy by downregulation of miR-21. It has been suggested that miR-21 confers chemoresistance in cancer cells by regulating the expression of phosphatase and tensin homolog (PTEN) and programmed cell death 4 (PD4D4) [27, 28, 30], and berberine sensitizes cancer cells through PTEN/Akt signaling pathway [22]. Moreover, it was reported that berberine improves the therapeutic efficiency of cisplatin through miR-21/PDCD4 axis [45]. As such, it is possible that PTEN and PD4D4 are involved in the chemosensitizing effect. In addition, it was reported that berberine suppresses multiple myeloma cell growth by reducing miR-21 through IL6/Stat3 regulation [31]. Further investigation is needed to unravel the detailed mechanism underlying the association between berberine and miR-21 in chemosensitizing effect of OSCC.

In conclusion, we showed the anti-CSC effect of berberine on oral cancer via targeting miR-21, therefore leading to reduction of self-renewal and metastatic properties *in vitro* and attenuation of tumor growth *in vivo*. Our findings provided the evidence for using the natural berberine as an adjunctive therapy to traditional chemotherapeutics.

## MATERIALS AND METHODS

### Reagent and cell culture

Berberine was purchased from Sigma-Aldrich Chemical Co. (St. Louis, MO, USA) Berberine was further diluted in culture medium to the appropriate final concentrations prior to use. The CSCs derived from OSCC cell lines SAS and OECM-1 as well as primary normal human gingival epithelioid cell line (SG) were cultivated as previously described [34].

### Cell viability assay

Cytotoxicity of berberine was determined by MTT (Sigma, St. Louis, MO) assay. Cells were seeded in 24-well plates (1 × 10^4^ cells/ well) in the presence of various concentration of berberine or vehicle at 37°C for 24 hours followed by incubation with MTT reagent. After removing the supernatant, blue formazan crystals of viable cells were dissolved in DMSO and evaluated spectrophotometrically at 570 nm. DMSO-treated group was set as 100%, and data were presented as percentage of DMSO control.

### Aldefluor™ assay

ALDH1 enzymatic activity was detected using the Aldefluor™ kit (Stem Cell Technologies Inc., Vancouver, Canada) according to the manufacturer's instructions. Cells stained with a specific ALDH inhibitor, 4-diethylaminobenzaldehyde (DEAB), served as negative control. Fluorescence emission from 10,000 cells was assessed with FACSCalibur (Becton Dickinson, Mountain View, CA, USA) using CellQuest software

### Secondary sphere formation assay

Cells were dissociated and cultured in the modified DMEM/F-12 supplemented with N2 (R&D Minneapolis, MN, USA), 10 ng/mL epidermal growth factor (Invitrogen, Carlsbad, CA, USA), 10 ng/mL basic fibroblast growth factor (Invitrogen) and penicillin/streptomycin at 10^4^ live cells/low-attachment six-well plate (Corning Inc., Corning, NY, USA) along with various concentration of berberine. Cell density/ 10,000 cells were calculated and shown as percentage of control group.

### Soft agarose assay

Each well of a six-well culture dish was coated with 1 ml of bottom agar (Sigma-Aldrich) mixture (DMEM/F-12, 15% (v/v) FBS, 0.525% (w/v) agar). After the bottom layer was solidified, 1 ml of top agar-medium mixture (DMEM/F-12, 15% (v/v) FBS, 0.3% (w/v) agar) containing 4 × 10^4^ cells was added, and the dishes were incubated at 37°C for 2 weeks. Plates were stained with 0.01% crystal violet, and then the colonies were counted.

### Cell invasion and migration assays

The 24-well plate Transwell system with a polycarbonate filter membrane of 8-μm pore size (Corning, United Kingdom) was used to evaluate the migration and invasion abilities of cells. The membrane was coated with Matrigel (BD Pharmingen, NJ, USA) for invasion. The cell suspensions were seeded to the upper chamber of the Transwell insert within serum-free medium at the cell density of 5 × 10^4^ and 1 × 10^5^ for migration and invasion assays, respectively. The lower chamber was filled with media supplemented with 10% serum and various concentration of berberine. After 24 hours of incubation, the filter membrane was stained with crystal violet. The migrated and invasion cells were counted from five different visual areas of 100-fold magnification under an inverted microscope.

### Measurement of tumor growth *in vivo*

All procedures involving animals were conducted in accordance with the institutional animal welfare guidelines of the Chung Shan Medical University. 5–6 weeks old immuno-deficient nude mice (BALB/c nu/nu mice) were used for the xenograft model. OCSC (1 × 10^4^ cells/0.2 mL/mouse) were injected subcutaneously into the right axilla and the day of cell implantation was designated as day 0. Mice were randomly divided into two groups and fed with either placebosaline (control) or berberine (10 and 20 mg/kg per day) by oral gavage. Tumor size measurement was performed using an IVIS50 animal imaging system (Xenogen Corp.). The volume was calculated (according to the following formula: [length × width^2^]/2), and then analyzed by Image-Pro Plus software. After 23 days, animals were euthanized followed by tissue excision for phosphor-Stat3 analysis.

### miR-21 down-regualtion

miR-21 inhibitor, and scramble (Scr) control were purchased from Applied Biosystems (Foster City, CA, USA). LipofectamineTM 3000 transfection reagent (Invitrogen, Thermo Fisher Scientific Inc., Carlsbad, CA, USA) according to the manufacturer's protocol was used as the transfection reagent.

### Statistical analysis

SPSS software (version 13.0; SPSS, Inc., Chicago, IL, USA) was used for statistical analysis. The presented results are representative of three independent experiments with similar results. Statistical differences were evaluated with the Student *t* test, and *p* < 0.05 was considered significant.
