# Correction: Berberine-targeted miR-21 chemosensitizes oral carcinomas stem cells

**DOI:** 10.18632/oncotarget.25460

**Published:** 2018-05-15

**Authors:** Che-Yi Lin, Pei-Ling Hsieh, Yi-Wen Liao, Chih-Yu Peng, Ming-Yi Lu, Ching-Hsuan Yang, Cheng-Chia Yu, Chia-Ming Liu

**Affiliations:** ^1^ Department of Oral and Maxillofacial Surgery, Chi Mei Hospital, Tainan, Taiwan; ^2^ Institute of Oral Sciences, Chung Shan Medical University, Taichung, Taiwan; ^3^ School of Dentistry, Chung Shan Medical University, Taichung, Taiwan; ^4^ Department of Dentistry, Chung Shan Medical University Hospital, Taichung, Taiwan

**This article has been corrected:** The correct 4th affiliation is given below:

^**4**^ Department of Dentistry, Chung Shan Medical University Hospital, Taichung, Taiwan

Original article: Oncotarget. 2017; 8:80900-80908. https://doi.org/10.18632/oncotarget.20723

